# Upregulated expression of LncRNA nicotinamide nucleotide transhydrogenase antisense RNA 1 is correlated with unfavorable clinical outcomes in cancers

**DOI:** 10.1186/s12885-020-07348-5

**Published:** 2020-09-14

**Authors:** Chenghao Zhang, Xiaolei Ren, Zhongyue Liu, Chao Tu

**Affiliations:** 1 Department of Orthopedics, The Second Xiangya Hospital, Central South University, Changsha, Hunan 410011 PR China; 2 Hunan Key Laboratory of Tumor Models and Individualized Medicine, The Second Xiangya Hospital, Central South University, Changsha, Hunan 410011 PR China

**Keywords:** LncRNA, NNT-AS1, Cancer, Sarcoma, Prognosis

## Abstract

**Background:**

The nicotinamide nucleotide transhydrogenase antisense RNA 1 (NNT-AS1) is a long non-coding RNA aberrantly expressed in human malignancies. We aimed to analyze available data to evaluate the correlation between NNT-AS1 expression and cancer prognosis.

**Methods:**

Literature retrieval was performed by systematic searching related databases from inception to April 2, 2020. Studies regarding correlation between NNT-AS1 expression, survival outcomes and clinical characteristics of cancer patients were collected and pooled to calculate the the hazard ratios (HRs) or odds ratios (ORs) with 95% confidence intervals (95% CIs).

**Results:**

Ten studies comprising 699 patients were included, all of which were conducted in China according to literature selection criteria. Overexpression of NNT-AS1 had a significant association with unfavorable overall survival (OS) (HR = 2.08, 95% CI: 1.84–2.36, *P* < 0.001). Stratified analysis showed that tumor type, sample size, follow-up months, and survival analysis approach did not change the predictive value of NNT-AS1 on OS. Furthermore, elevated NNT-AS1 level had significant association with distant metastasis (DM) (OR = 2.45, 95% CI: 1.39–4.30), lymph node metastasis (LNM) (OR = 3.92, 95% CI: 1.35–11.41), TNM stage (OR = 4.25, 95% CI: 1.71–10.56), and vascular invasion (OR = 3.98, 95% CI: 2.06–7.71), but was not associated with age and gender. The TCGA dataset further consistently showed that the NNT-AS1 expression was associated with poor OS and disease-free survival.

**Conclusions:**

High expression of NNT-AS1 is associated with unfavorable survival outcomes and poor clinicopathologic characteristics. However, large-cohort data and geographical studies are still needed to further validate the prognostic value of NNT-AS1 in cancers.

## Background

Cancer has become a global health burden and posed a threat to human development over the past decades [1, 2]. Due to cancer, there were 17.2 million incident malignancy cases, 8.9 million deaths, and 213.2 million disability-adjusted life-years worldwide in 2016. Notably, incident cases increased by 28%, of which the largest increase occurred in the least developed countries between 2006 and 2016 [3]. Though tremendous achievements have been made in surgery, chemotherapy, targeted therapy, and the recent immunotherapy in the past years [4, 5], the prognosis of cancer patients still remains poor, which may be ascribed to the lack of effective predictive factors in malignancies. Thus, many investigators have been endeavored to explore novel putative biomarkers for predicting prognosis and therapeutic efficacy in cancer patients [6].

Long non-coding RNAs (lncRNAs) belong to non-coding RNAs whose lengths are longer than 200 bp [7]. They have little or no capability of coding protein [8]. Recent studies have demonstrated that lncRNAs could drive pathophysiologic phenotypes through interaction with other cellular macro-molecules including DNA, RNA and proteins [9]. Aberrant expression or functional abnormalities of LncRNAs have been linked with numerous human diseases, such as aging [10], degenerative disease [11], and cancer [7, 12–14]. Recently, a pivotal role of lncRNA in tumor biological characteristics including proliferation, cell cycle arrest, invasion, migration [15], autophagy [16], and drug resistance [17] has also been revealed.

Nicotinamide nucleotide transhydrogenase antisense RNA 1 (NNT-AS1) is a newly identified lncRNA located in the chromosome 5p12 region with three exons, and transcribed in the opposite direction of NNT [18]. Emerging studies have demonstrated that NNT-AS1 could play a crucial role in carcinogenesis, and aberrant expression of NNT-AS1 was significantly associated with survival outcome in various cancers. However, most individual studies evaluating NNT-AS1 expression in cancers remain unconvincing as a result of the limitations in small sample size and possible controversial outcomes. For example, although all eligible studies reported that elevated NNT-AS1 expression was correlated with worse overall survival (OS), study design characteristics such as tumor types, follow-up months, and cut-off values were different among studies. Besides, heterogeneous outcomes regarding clinicopathologic parameters also existed among included studies. For instance, high expression of NNT-AS1 was associated with unfavorable tumor node metastasis (TNM) stage in most studies, but not in study performed by Li Y et al. [19]. Both Ye H et al. [15]*,* and Gu Y et al. [20] reported that upregulated NNT-AS1 indicated positive distant metastasis (DM), but another study conducted by Wang Q et al. [21] showed inconsistent results. Giving the discrepancies and potential underpowered statistical efficacy among those studies, we firstly conducted this comprehensive meta-analysis to elucidate the predictive value of NNT-AS1 in cancer prognosis. This study aimed to provide an objective evaluation on heterogeneity among included studies, and demonstrate the functional mechanisms of NNT-AS1 in carcinogenesis.

## Methods

### Searching strategy

We searched potential literature MEDLINE, the Cochrane Library, Web of Science, Embase, Scopus, and China National Knowledge Infrastructure (CNKI) database from their inception up to April 2, 2020 to locate articles. In order to strengthen the searching sensitivity, both MeSH terms and free-text words were used. The terms were listed as follows: (“nicotinamide nucleotide transhydrogenase antisense RNA 1” or “NNT-AS1”) AND (“carcinoma” or “sarcoma” or “cancer” or “tumor” or “neoplasm” or “malignancy”) with the limit to human. An additional manual search of citation lists of retrieved literature was performed. Of note, the present study was critically projected, reviewed and reported on the basis of the PRISMA checklist to enhance the credibility of the results [22].

### Study selection

For inclusion in the present meta-analysis, the studies should met the following criteria: 1) articles investigating the association between NNT-AS1 expression level and survival outcome in human cancers; 2) patients were categorized into two groups based on the expression of NNT-AS1; 3) patients were diagnosed with cancer by histopathological examination; 4) sufficient original data for extracting or calculating the individual hazard ratios (HRs)/odds ratios (ORs) with its 95% CIs; 5) related clinicopathologic parameters including lymph node metastasis (LNM) and DM were described.

By contrast, studies were excluded according to the following criteria: 1) literature irrelevant to cancer or NNT-AS1; 2) duplicate publications; 3) studies lack of usable clinical data, including animal experiments and those about the structure or functions of NNT-AS1; and 4) letters, editorial, abstracts, case reports or reviews.

### Data extraction

All data elements in the enrolled studies were rigorously assessed and extracted by two independent investigators (CT and CHZ), and disagreements were resolved through discussion or consultation from the third investigator (XLR). We extracted the following data from included studies: surname of first author, year of publication, country of origin, tumor type, total number of patients, patients` number in high NNT-AS1 expression group and low NNT-AS1 expression group, clinicopathologic features, detection and survival analysis method, cut-off value, HRs with corresponding 95% CIs regarding to OS, progression-free survival (PFS) or disease-free survival (DFS).

If the data was unavailable, we contacted the corresponding author of original article to request the missing data. When only Kaplan-Meier curves were available in certain studies, the survival rates were indirectly extracted from the graphical plots and calculated HRs with 95% CIs were determined via Engauge Digitizer software (Version 4.1) as previously described [23].

### Quality assessment

Two investigators (ZYL and XLR) evaluated the quality of eligible studies independently according to the Newcastle-Ottawa Scale (NOS). Generally, the studies with NOS score ≥ 7 were considered to be of high methodological quality [24] .

### Public data and tools

This study is consistent with the publication guidelines provided by The Cancer Genome Atlas (TCGA). TCGA Data portal (https://portal.gdc.cancer.gov) was applied into extracting the clinical data as well as RNAseqV2. Gene Expression Profiling Interactive Analysis (GEPIA) was applied for analysis of the data as described previously [25]. Differential expression analysis was carried out via one-way ANOVA. While the survival analysis, including OS and DFS, were performed by Kaplan–Meier (K-M) and log-rank test, and HRs and *p*-value were presented as K-M curves.

### Statistical methods

All statistical analyses were performed via STATA software (Version 12.0) and Review Manager (RevMan 5.3). Pooled HR with 95% CI was extracted from included studies. The Log HR and standard error (SE) were applied for aggregation of the survival outcomes. Heterogeneity across all studies was determined by *I*^*2*^ statistics and chi-squared test. If *I*^*2*^ > 50% or the chi-squared test shows *p* < 0.10, which represented significant heterogeneity among the studies, the random-effects model was applied for analysis. In contrary, if apparent between-study heterogeneity was not observed (*p* > 0.10 and *I*^*2*^ < 50%), the fixed-effects model was adopted.

Sensitivity analysis of NNT-AS1 expression was conducted by sequentially omitting individual study to verify the stability of outcomes in this meta-analysis. The potential publication bias was estimated via Begg’s funnel plot and Egger’s test. If the funnel plot showed asymmetry or Egger’s test showed *P* < 0.05, the publication bias was considered to be statistically significant.

## Results

### Characteristics of eligible studies

**Fig. 1:**
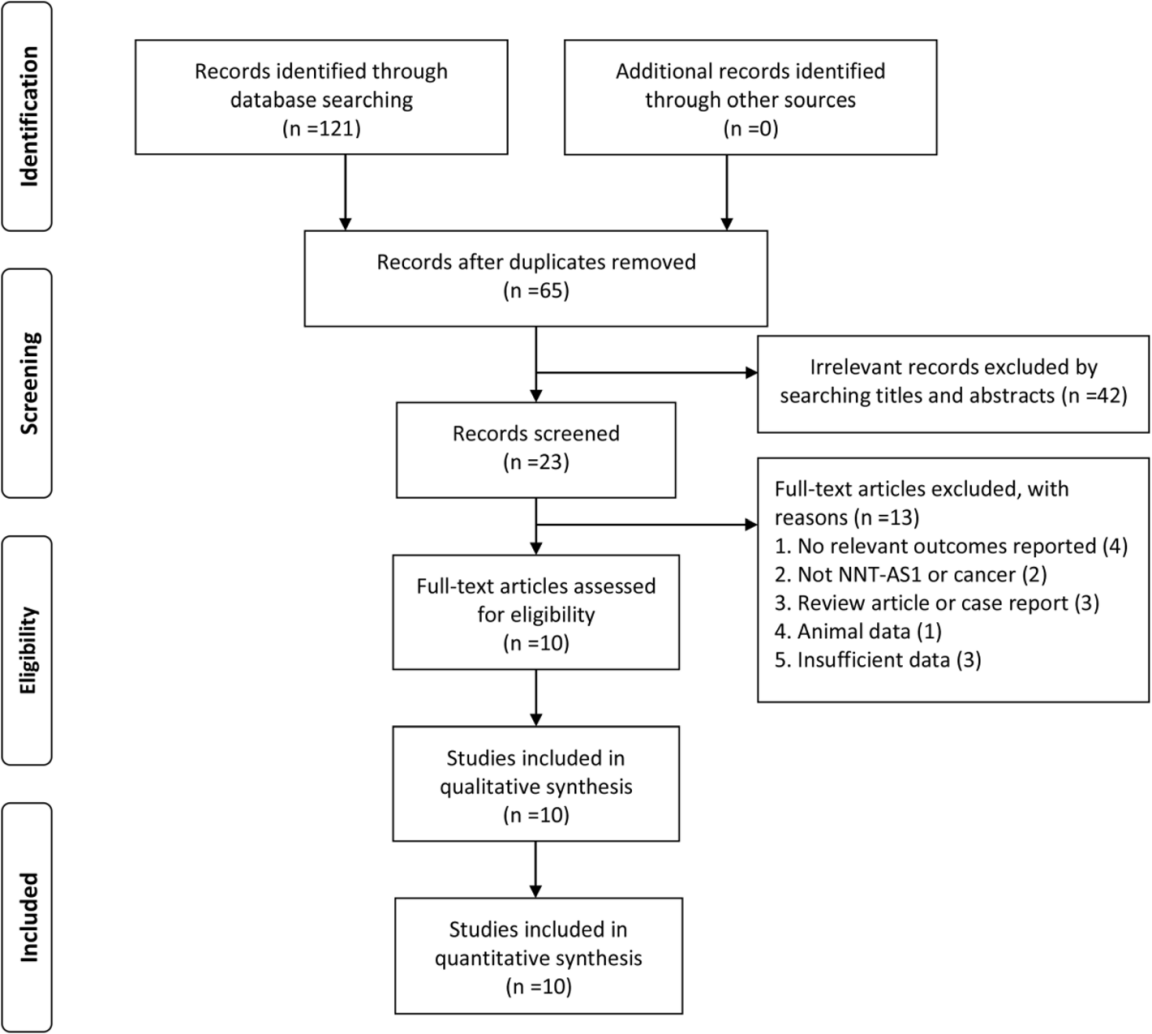
Flow diagram of study selection procedure

This study was conducted following the PRISMA Checklist, as shown in Table S1. A total of 121 studies were initially identified as potential articles. After removing the duplications, 65 studies were screened through titles and abstracts. Afterwards, 42 articles with irrelevant records were excluded. The remaining 23 full-text articles were further evaluated. Thirteen studies were excluded as a result of irrelevant topics, insufficient data, animal study, and review/ case report. Finally, ten articles compromising 699 patients were included to carry out qualitative and quantitative synthesis. As demonstrated in Fig. 1, the selection procedure was presented by a flow diagram.

The main characteristics of the included studies were demonstrated in Table 1. These articles were published between 2017 and 2019 with a sample size ranging from 42 to 126. In all eligible studies, NNT-AS1 gene expression experiments in this meta-analysis were carried out on primary tumors collected from patients. Generally, the enrolled patients were distributed in two groups (high or low NNT-AS1 expression), considering the levels of NNT-AS1 as measured by qRT-PCR. All of these investigations were carried out in China. Eight divergent types of cancers were analyzed in our meta-analysis, including osteosarcoma, breast cancer, gastric cancers, bladder cancer, cholangiocarcinoma, hepatocellular carcinoma, colorectal cancer and cervical cancer. The follow-up months for survival outcome ranged from 39 to 80 months. Seven studies adopted univariate analysis for the survival analysis method and the other three articles performed multivariate analysis. Furthermore, these studies also investigated other clinicopathologic parameters, such as age, gender, clinical stage, vascular invasion, LNM and DM. As to clinical stage, it should be noted that most studies adopted the TNM classification system, while two studies used the Enneking [15] or the International Federation of Gynecology and Obstetrics (FIGO) staging [30]. All of these eligible studies are of high quality with a NOS score ≥ 7. Details of the NOS scoring were reported in the supplementary file (Table S2).

**Table 1:** Summary of the main characteristics of the studies enrolled in the meta-analysis

Study	Year	Country of origin	Tumor type	Sample size	NNT-AS1 expression		Follow-up months	Detection assay	Clinical stage	Metastasis analysis	Outcome measure	Survival analysis	Cut-off value	NOS	Ref.
					High	Low									
Gu, Y et al	2019	China	Cholangiocarcinoma	98	N/A	N/A	60	qRT-PCR	TNM I-III	DM	OS	Multivariate	Mean	8	[20]
Huang, L et al	2019	China	Cholangiocarcinoma	48	27	21	36	qRT-PCR	TNM I-IV	LNM	OS	Univariate	Median	8	[26]
Wu, D et al	2019	China	Bladder cancer	47	24	23	60	qRT-PCR	TNM I-IV	LNM	OS	Univariate	Median	8	[27]
Chen, B et al	2018	China	Gastric cancer	48	27	21	60	qRT-PCR	TNM I-IV	LNM	OS	Univariate	Median	7	[28]
Gu, Y et al	2018	China	Gastric cancer	77	39	38	65	qRT-PCR	TNM I-IV	LNM	OS	Univariate	Mean	8	[29]
Ye, H et al	2018	China	Osteosarcoma	126	63	63	80	qRT-PCR	Enneking IA-III	DM	OS/DFS	Univariate	Median	7	[15]
Li, Y et al	2018	China	Breast cancer	64	32	32	60	qRT-PCR	TNM I-III	N/A	OS	Multivariate	Mean	8	[19]
Lu, Y et al	2017	China	Hepatocellular cancer	42	23	19	50	qRT-PCR	TNM I-IV	LNM	OS	Univariate	Median	7	[18]
Wang, Q et al	2017	China	Colorectal cancer	70	35	35	39	qRT-PCR	TNM I-IV	LNM/DM	OS/PFS	Multivariate	Median	9	[21]
Hua, F et al	2017	China	Cervical cancer	79	40	39	60	qRT-PCR	FIGO Ib-IIIa	LNM	OS	Univariate	Median	8	[30]

*DFS* disease-free survival, *DM* distant metastasis, *FIGO* the International Federation of Gynecology and Obstetrics, *HR* hazard ratio, *LNM* lymph node metastasis, *N/A* not available, *NNT-AS1* nicotinamide nucleotide transhydrogenase antisense RNA 1, *NOS* Newcastle-Ottawa Scale, *OS* overall survival, *PFS* progression-free survival, *TNM* tumor node metastasis

### Association between NNT-AS1 and OS

**Fig. 2:**
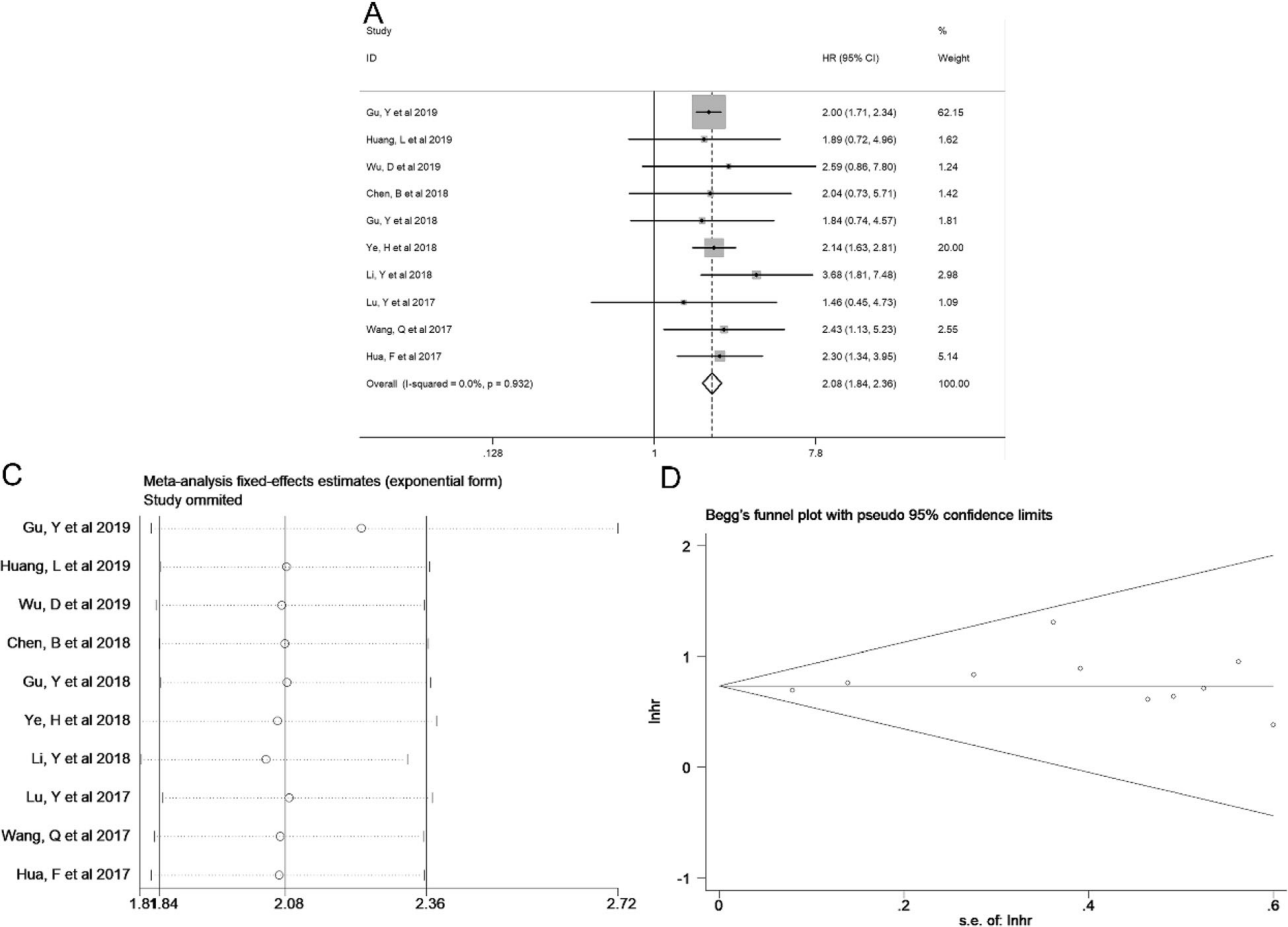
**a** Forest plot of studies evaluating the relationship between NNT-AS1 and OS, **b** sensitivity analysis for OS, and **c** Begg’s publication bias plots of OS. NNT-AS1: nicotinamide nucleotide transhydrogenase antisense RNA 1; OS: overall survival

We used fixed-effects model to analyze the pooled HR and corresponding 95% CI since heterogeneity among these studies was not obvious (*I*^2^ = 0.0%, *p* = 0.932). As presented in Fig. 2a, the pooled result showed that high expression of NNT-AS1 predicted unfavorable OS in cancers (HR = 2.08, 95% CI: 1.84–2.36, *P* < 0.001).

**Fig. 3:**
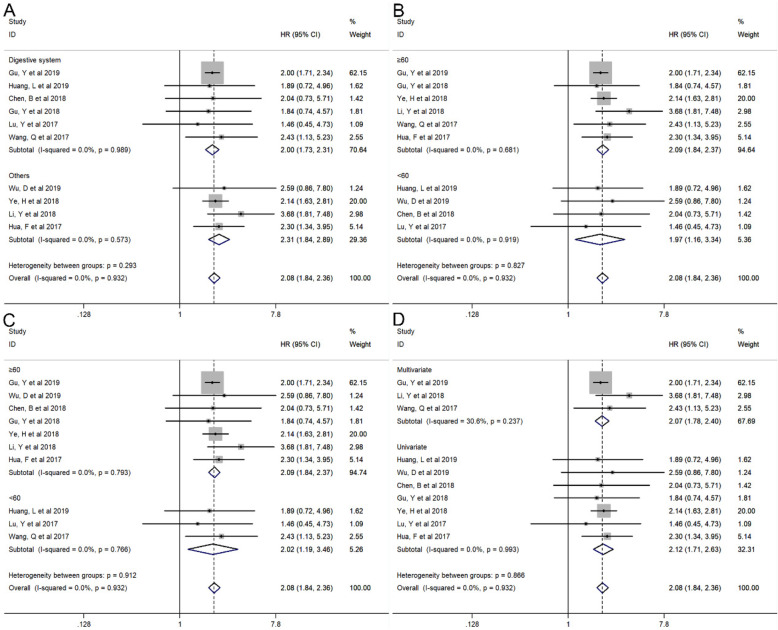
Forest plots evaluating the stratified analyses of NNT-AS1 expression with **a** tumor type, **b** sample size, **c** follow-up months and **d** survival analysis method. NNT-AS1: nicotinamide nucleotide transhydrogenase antisense RNA 1

In addition, stratified analyses were performed to investigate the relevance between NNT-AS1 expression with OS in different subgroups according to tumor type (digestive system or others), sample size (more or less than 60), follow-up months (more or less than 60), and survival analysis method (univariate or multivariate analysis). The results revealed that all stratified analyses recapitulated the predictive potential of NNT-AS1 for OS in malignancies (Fig. 3 and Table 2).

**Table 2:** Stratified analyses of the pooled HRs of overall survival by tumor type, sample size, follow-up months, and survival analysis method

Subgroup analysis	No. of studies	No. of patients	Pooled HR (95% CI)		Heterogeneity	
			Fixed model	*p*-value	*I*^2^ (%)	*p*-value
Tumor type						
Digestive system	6	374	2.00 (1.73, 2.31)	0.000	0.0	0.989
Others	4	316	2.31 (1.84, 2.89)	0.000	0.0	0.573
Sample size						
≥60	6	505	2.09 (1.84, 2.37)	0.000	0.0	0.932
< 60	4	185	1.97 (1.16, 3.34)	0.012	0.0	0.681
Follow-up months						
≥60	7	530	2.09 (1.84, 2.37)	0.000	0.0	0.793
< 60	3	160	2.02 (1.19, 3.46)	0.01	0.0	0.766
Survival analysis method						
Multivariate	3	223	2.07 (1.78, 2.40)	0.000	30.6	0.237
Univariate	7	467	2.12 (1.71, 2.63)	0.000	0.0	0.993

*CI* confidence interval, *HR* hazard ratio

### Association between NNT-AS1 and other clinicopathologic parameters

**Fig. 4:**
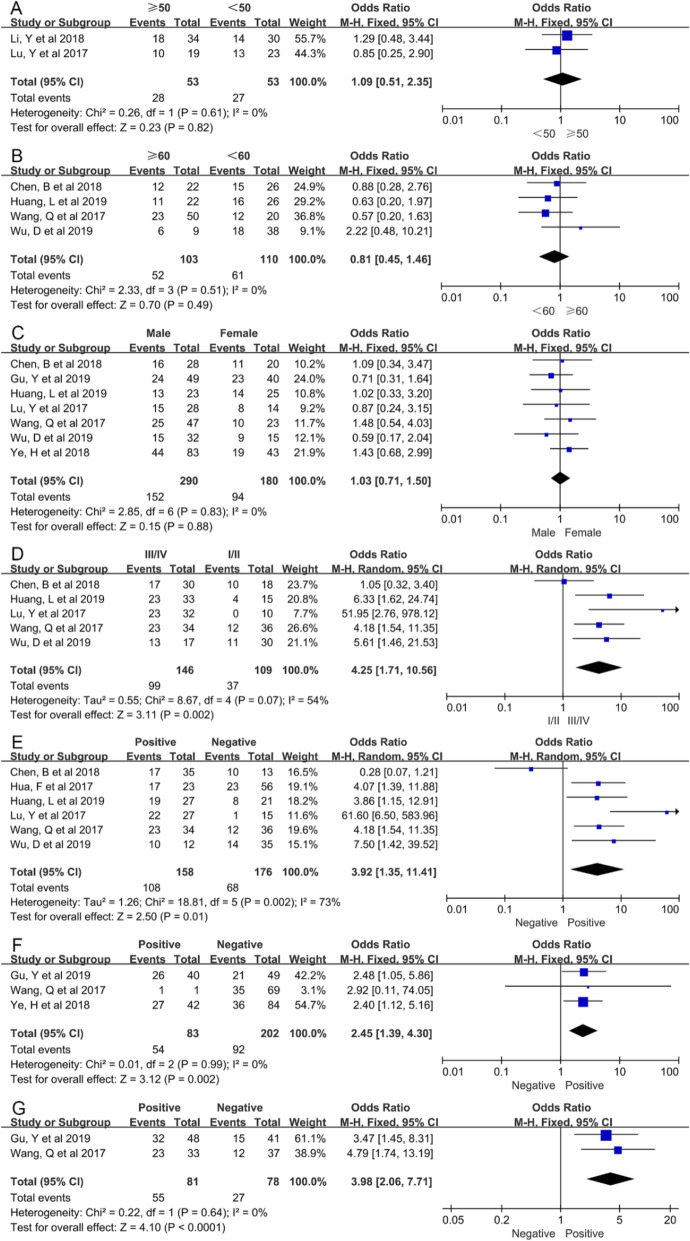
Forest plots of published articles evaluating the relationship between NNT-AS1 expression and other clinicopathologic features, including **a** age (≥50/< 50), **b** age (≥60/< 60), **c** gender, **d** clinical stage, **e** LNM, **f** DM, and **g** Vascular invasion. DM: distant metastasis; LNM: lymph node metastasis; NNT-AS1: nicotinamide nucleotide transhydrogenase antisense RNA 1

In addition, ORs with corresponding 95% CIs were applied to detect the association between NNT-AS1 and other clinicopathological parameters. The results of these analyses were summarized in Fig. 4 and Table 3. Notably, fixed-effects model was applied in analyzing the association between NNT-AS1 and several clinicopathologic characteristics including age, gender, vascular invasion, and DM, since no obvious heterogeneity was observed. High expression of NNT-AS1 was significantly correlated to vascular invasion (OR = 3.98, 95% CI: 2.06–7.71) and DM (OR = 2.45, 95% CI: 1.39–4.30), but not age and gender.

**Table 3:** Correlation between lncRNA NNT-AS1 expression and other clinicopathological parameters for cancers

Clinicopathologic parameters	No. of Studies	No. of Participants	Pooled OR (95% CI)	*P*	Model	Heterogeneity
						Chi^2^, *p-value*, *I*^*2*^ (%)
Age (≥50/< 50)	2	106	1.09 (0.51, 2.35)	0.82	Fixed	0.26, 0.61, 0
Age (≥60/< 60)	4	213	0.81 (0.45, 1.46)	0.49	Fixed	2.33, 0.51, 0
Gender (Male/Female)	7	470	1.03 (0.71, 1.50)	0.88	Fixed	2.85, 0.83, 0
TNM stage (III-IV/I-II)	5	255	4.25 (1.71, 10.56)	0.002	Random	8.67, 0.07, 54
LNM (Positive/Negative)	6	334	3.92 (1.35, 11.41)	0.01	Random	18.81, 0.002, 73
DM (Positive/Negative)	3	285	2.45 (1.39, 4.30)	0.002	Fixed	0.01, 0.99, 0
Vascular invasion (Positive/Negative)	2	159	3.98 (2.05, 7.71)	*P* < 0.0001	Fixed	0.22, 0.64, 0

*CI* confidence interval, *DM* distant metastasis, *LNM* lymph node metastasis, *OR* odds ratio, *NNT-AS1* nicotinamide nucleotide transhydrogenase antisense RNA

By contrast, the random-effects model was used to analyze the correlation between NNT-AS1 and clinical characteristics including clinical stage and LNM due to the apparent between-study heterogeneity. Significantly, upregulated expression of NNT-AS1 predicted worse clinical stage (OR = 4.25, 95% CI: 1.71–10.56) and LNM (OR = 3.92, 95% CI: 1.35–11.41).

### Sensitivity analysis and publication bias

In order to assess the stability of the aforementioned results, sensitivity analysis was performed. When each eligible study was removed, the result of NNT-AS1 for OS was not obviously changed, indicating the conclusion is reliable (Fig. 2b).

Besides, the publication bias, regarding correlation between expression level of NNT-AS1 and OS, was evaluated via conducting Begg’s funnel plot and Egger’s regression test. The Begg’s funnel plot was symmetry, and Egger’s test showed *P* = 0.369, suggesting no obvious publication bias was measured (Fig. 2c).

### Validation of the result1s in TCGA dataset

**Fig. 5:**
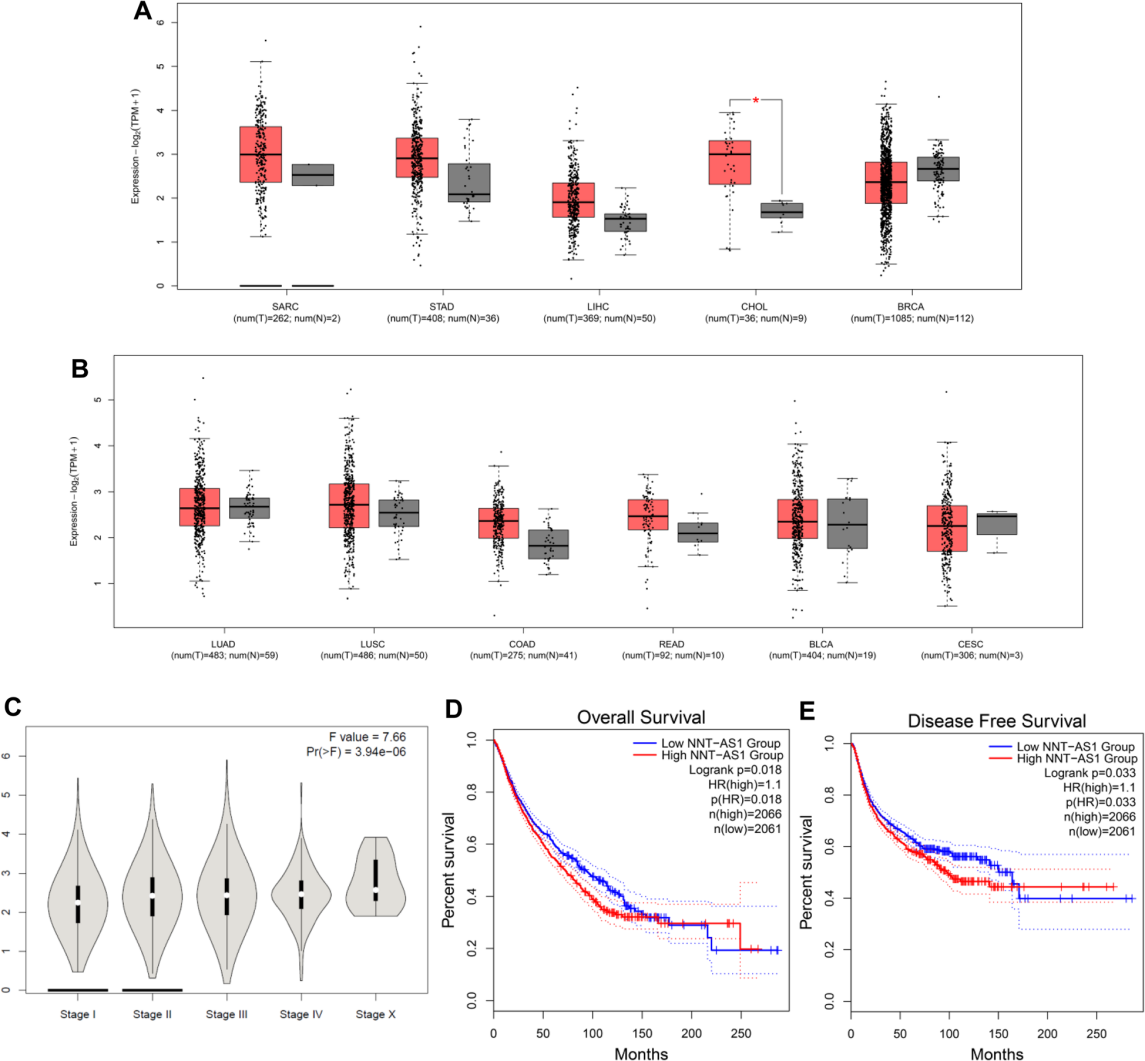
Validation of NNT-AS1 expression in various cancers in TCGA cohort. **a** The expression levels of NNT-AS1 in SARC, STAD, LIHC, CHOL, and BRCA. **b** The expression levels of NNT-AS1 in LUAD, LUSC, COAD, READ, BLCA, and CESC. **c** Association between NNT-AS1 expression and clinical stage of cancers including SARC, STAD, LIHC, CHOL, COAD, BRCA, LUAD, LUSC, COAD, READ, BLCA, and CESC in TCGA cohort. **d** OS plot of NNT-AS1 in TCGA cohort (*n* = 4127). **e** DFS plot of NNT-AS1 in TCGA cohort (*n* = 4127). BLCA (bladder urothelial carcinoma); BRCA (breast invasive carcinoma); CESC (cervical squamous cell carcinoma and endocervical adenocarcinoma); CHOL (cholangiocarcinoma); COAD (colon adenocarcinoma); DFS: disease-free survival; LIHC (liver hepatocellular carcinoma); LUAD (lung adenocarcinoma); LUSC (lung squamous cell carcinoma); NNT-AS1: nicotinamide nucleotide transhydrogenase antisense RNA 1; OS: overall survival; READ (rectum adenocarcinoma); SARC (sarcoma); STAD (stomach adenocarcinoma); TCGA: the Cancer Genome Atlas

Furthermore, the expression levels of NNT-AS1 in related cancers were explored by utilizing the data originated from TCGA. As demonstrated in Fig. 5, NNT-AS1 showed significantly elevated expression in cholangiocarcinoma when compared with control. Besides, NNT-AS1 was aberrantly expressed in sarcoma, stomach adenocarcinoma, liver hepatocellular carcinoma, colon adenocarcinoma, and rectum adenocarcinoma, but the differences were not significant. Moreover, NNT-AS1 expression level was markedly correlated with clinical stage in human cancers. In addition, we merged the expression data and OS/DFS data of carcinomas from TCGA dataset derived from GEPIA, which comprising 4127 patients categorized in high or low expression group, including BLCA (bladder urothelial carcinoma), CESC (cervical squamous cell carcinoma and endocervical adenocarcinoma), BRCA (breast invasive carcinoma), CHOL (cholangiocarcinoma), COAD (colon adenocarcinoma), LIHC (liver hepatocellular carcinoma), LUAD (lung adenocarcinoma), LUSC (lung squamous cell carcinoma), READ (rectum adenocarcinoma), SARC (sarcoma), STAD (stomach adenocarcinoma). These results suggested that the upregulated NNT-AS1 expression predicted worse OS (HR = 1.1, *p* = 0.018), as well as DFS (HR = 1.1, *p* = 0.033), confirming that overexpression of NNT-AS1 was significantly correlated to unfavorable survival outcomes in cancer patients.

## Discussion

Recently, emerging studies have explored the possible link between expression of lncRNA NNT-AS1 and human tumors. Compared with adjacent noncancerous tissue and normal cell, upregulated NNT-AS1 expression was identified in most cancer tissues or cell lines and therefore indicated poor survival outcome, such as osteosarcoma [31], breast cancer [19], cervical cancer [30], gastric cancer [28], hepatocellular carcinoma [18], colorectal cancer [21], and non-small cell lung cancer (NSCLC) [2]. On the contrary, another study performed by Huang et al, claimed that NNT-AS1 was markedly downregulated in patients with ovarian cancer and ovarian cell lines [32]. However, results from above-mentioned studies should be interpreted with caution because of the limited sample size and discrete outcomes. Therefore, we designed and carried out this meta-analysis to further elucidate the correlation between NNT-AS1 and clinicopathologic outcomes and prognostic values in cancers.

Ten studies with eight cancer types containing 699 patients were pooled together in this study, and the results suggested that promoted NNT-AS1 expression was significantly associated with unfavorable prognosis of OS in patients with cancers. Subgroup stratified analysis further demonstrated that the tumor type, sample size, follow-up months, and survival analysis method did not alter the correlation between TTN-AS1 and OS. Thus, it seems like that differences among study design characteristics have no obvious effect on NNT-AS1 correlation with OS, suggesting that our pooled results were reliable. No publication bias regarding NNT-AS1 expression for OS was observed, indicating the credibility of our results. Furthermore, pooled data from TCGA dataset showed that NNT-AS1 was obviously correlated with OS (HR = 1.1, *p* = 0.018), which was consistent with our results. Since tumor recurrence is also an imporant prognosis parameter for cancer patients, we next explored the association between NNT-AS1 expression and DFS via TCGA datasets. We showed that NNT-AS1 overexpression was correlated with unfavorable DFS (HR = 1.1, *p* = 0.033) in the cancer types involved in this meta-analysis (Fig. 5e). Moreover, one study [15] included in our analysis also reported that an elevated expression of TTN-AS1 predicted poor DFS in gastric cancer. However, interpretation regarding DFS should be careful since relevant studies were comparatively limited, and large cohort datasets are still needed for further validation the relationship between NNT-AS1 and tumor recurrence or other parameters. In addition, elevated NNT-AS1 level dramatically predicted worse clinical stage, vascular invasion, LNM, and DM. No significant association between NNT-AS1 and other clinicopathologic parameters including age and gender. Thus, even though NNT-AS1 expression has divergent association with clinicopathological parameters among included studies, our results still provided a reliable correlation via comprehensive meta-analysis. Consistent with our findings, result from the TCGA indicated that NNT-AS1 expression was significantly associated with clinical stage of human cancers. Taken together, our study firstly clarified the relationship between NNT-AS1 and cancer prognosis with a comprehensive evaluation on study heterogeneity and bias. TCGA dataset was explored to validate the role of NNT-AS1 in carcinomas, and the results showed that NNT-AS1 expression may serve as a potential indicator for cancer prognosis.

**Fig. 6:**
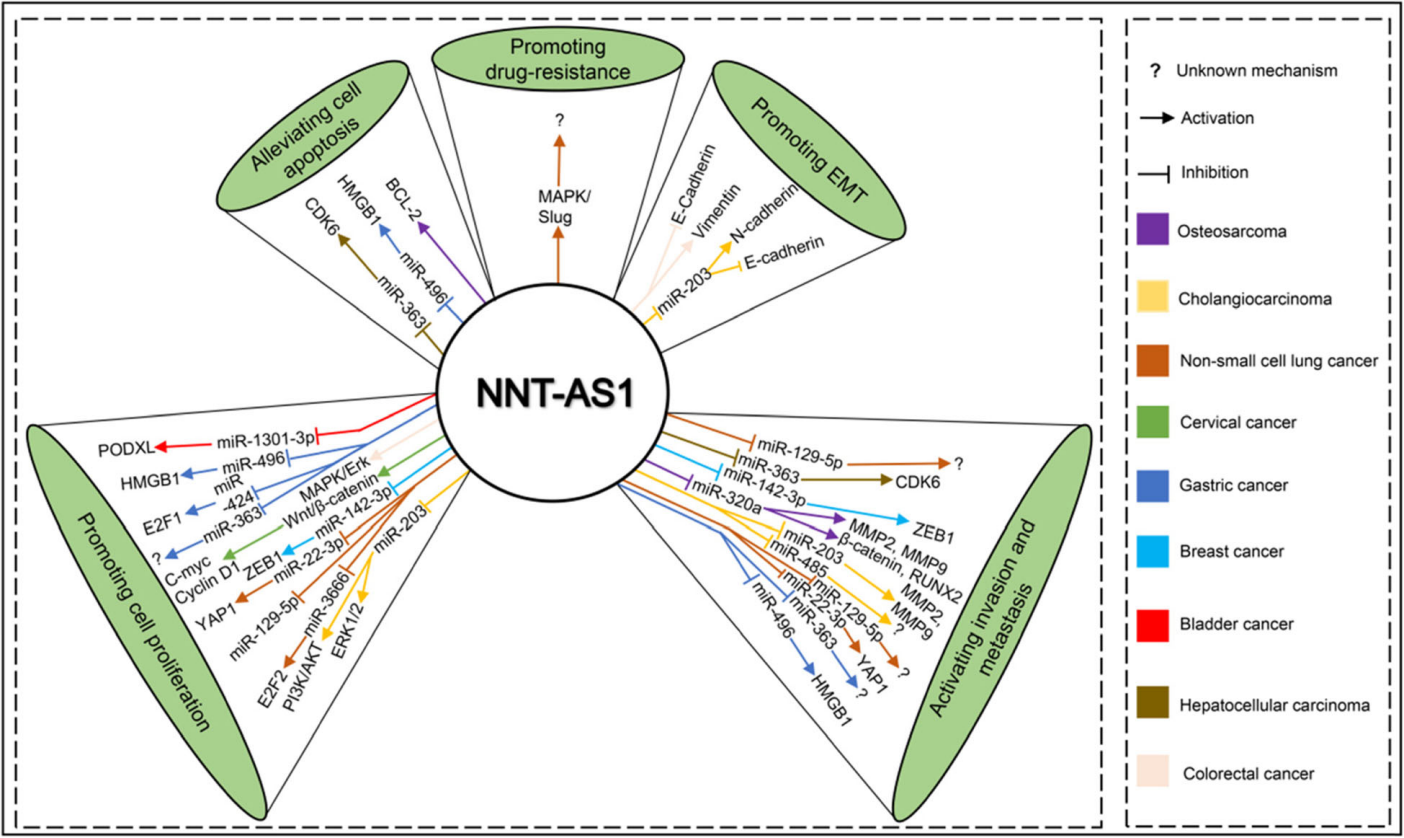
Schematic diagrams of various molecules and signaling pathways associated with NNT-AS1 in human cancers. Aberrant expression of NNT-AS1 was found in various cancers and dysregulation of NNT-AS1 contributed to carcinogenesis through different mechanisms, including promoting cell proliferation, alleviating cell apoptosis, activating invasion and metastasis, promoting EMT and drug-resistance. AKT: protein Kinase B; BCL-2: B-cell lymphoma 2; CDK6: cyclin-dependent kinase 6; E2F1: E2F Transcription Factor 1; EMT: epithelial–mesenchymal transition; ERK: extracellular-signal-regulated kinase; HMGB1: high mobility group box 1; MAPK: mitogen-activated protein kinase; MMP: matrix metalloproteinase; NNT-AS1: nicotinamide nucleotide transhydrogenase antisense RNA 1; PODXL: podocalyxin like; PI3K: phosphoinositide 3-kinase; RUNX2: runt-related transcription factor 2; YAP1: Yes-associated protein 1; ZEB1: zinc finger E-box-binding homeobox 1. Modified from Tamang S’s report [37]

Previous studies have investigated the underlying mechanisms of NNT-AS1 in carcinogenesis. Overexpression of NNT-AS1showed positive association with poorer OS, advanced tumor stage, LNM, depth of invasion [33], vessel invasion and differentiation in numerous cancers. Functional assays revealed that NNT-AS1 could promote proliferation, weaken cell cycle arrest and alleviate apoptosis by competing with CDK6 for miR-363 binding in hepatocellular carcinoma [18]. High expression of NNT-AS1 facilitates cholangiocarcinoma prognosis via promoting epithelial-mesenchymal transition (EMT) [20]. While knockdown or inhibition of NNT-AS1 could suppress cancer cell colony formation and invasion, arrested the cell cycle and promoted apoptosis both in vitro and in vivo [21]. Additionally, when silencing NNT-AS1 in colorectal cancer, EMT and MAPK/Erk pathway were inhibited [21]. Moreover, other pathways including PI3K/Akt/mTOR and Wnt/β-catenin signaling pathway were also found involved in the tumorigenesis and progression [15, 30]. Besides, NNT-AS1 was capable of serving as a competing endogenous RNA (ceRNA) by sponging miR-485/BCL9 or miR-203 in cholangiocarcinoma [26, 34], miR-1301-3p/PODXL or miR-496/HMGB1 in bladder cancer [3, 27], miR-142-3p/ZEB1 in breast cancer [19], miR-424/E2F1 or miR-363 in gastric cancer [8, 28], miR-22-3p/YAP1 or miR-129-5p in non-small cell lung cancer [33, 35], and miR-320a in osteosarcoma [31], therefore alteration in cancer cell function resulting from NNT-AS1 downregulation may be rescued by miRNA inhibition. Notably, NNT-AS1 also showed a high expression level in drug-resistant NSCLC, which promoted the cisplatin resistance of cancer cells via the MAPK/Slug pathway [36]. All these studies suggested that NNT-AS1 could serve as an oncogenic biomarker in cancer progression. The schematic diagram of various molecules and signaling pathways associated with NNT-AS1 in human cancers were displayed in Fig. 6.

Several deficiencies exist in this meta-analysis and they should be acknowledged. In the first place, our meta-analysis used the summarized data instead of raw data from the specific patients, and most of the HRs and 95% CIs were indirectly calculated by reconstructing survival curves instead of extracted from the original data, which inevitably could cause heterogeneity. Second, the cut-off value for NNT-AS1 expression differed across eligible studies due to the difficulty in reaching a consensus value, thus may introduce possible bias. Third, all enrolled studies were from China, which may cause biased results because of geographical differences. Thus, it should be cautious when applying our conclusions to the population in other regions. Fourth, data regarding NNT-AS1 expression levels with other prognostic outcomes, such as PFS, DFS were limited and thus unable to calculate the pooled value. Fifth, other factors such as different classification system of clinical stage, follow-up time, and analysis methods will also lead to possible bias. Sixth, the regulatory mechanisms of NNT-AS1 in cancer progression still remain largely unexplored. For instance, somatic mutation has been reported to affect lncRNA by regulation of methylation or expression of gene, miRNA, and transcriptional factor [38]. Currently, data regarding the role of somatic mutation on NNT-AS1 is lacking, which may merit future elucidation by using bioinformatics and in vitro assay [38, 39]. Last, in order to further clarify the correlation between NNT-AS1 expression with age or gender, more confounding variables, such as BMI, smoking history, or comorbidities, should be eliminated in order to minimize the possible bias [40]. Therefore, on the basis of the above limitations, well-designed comprehensive studies containing a large sample size, broader regions and countries, and more credible indicators are still warranted to further confirm our results.

## Conclusions

In summary, we found that overexpression of NNT-AS1 showed significant association with unfavorable survival outcomes and worse clinicopathological characteristics in kinds of human carcinomas. However, large-cohort data and geographical studies are still needed to conclude a prognostic role of NNT-AS1 as biomarker in cancer.

## Supplementary information

**Additional file 1: Table S1.** PRISMA Checklist.

**Additional file 2: Table S2.** Study quality and bias in the retrospective cohort studies judged by the Newcastle-Ottawa Scale (NOS) checklist.

## Data Availability

The data used and analyzed in the study is available from the corresponding author on reasonable request.

## Abbreviations

AKT: Protein Kinase B
BRCA: Breast invasive carcinoma
Bcl-2: B-cell lymphoma 2
CESA: Cervical squamous cell carcinoma and endocervical adenocarcinoma
CDK6: Cyclin-dependent kinase 6
COAD: Colon adenocarcinoma
E2F1: E2F Transcription Factor 1
EMT: Epithelial–mesenchymal transition
ERK: Extracellular-signal-regulated kinase
LUAD: Lung adenocarcinoma
LUSC: Lung squamous cell carcinoma
LIHC: Liver hepatocellular carcinoma
MAPK: Mitogen-activated protein kinase
MMP: Matrix metalloproteinase
mTOR: Mammalian target of rapamycin
NOS: Newcastle-Ottawa Scale
NNT-AS1: Nicotinamide nucleotide transhydrogenase antisense RNA1
OR: Odd ratio
OS: Overall survival
PI3K: Phosphoinositide 3-kinase
RUNX2: Runt-related transcription factor 2
READ: Rectum adenocarcinoma
SARC: Sarcoma
STAD: Stomach adenocarcinoma
TCGA: The Cancer Genome Atlas
YAP1: Yes-associated protein 1
ZEB1: Zinc finger E-box-binding homeobox 1
