# Supplementary material for: Upregulated expression of LncRNA nicotinamide nucleotide transhydrogenase antisense RNA 1 is correlated with unfavorable clinical outcomes in cancers
Source: BMC Cancer. 2020 Sep 14;20:879. doi: 10.1186/s12885-020-07348-5 (PMC7489002; doi:10.1186/s12885-020-07348-5)
Supplement: Supplementary file 2 — Additional file 2: Table S2. Study quality and bias in the retrospective cohort studies judged by the Newcastle-Ottawa Scale (NOS) checklist. [file 12885_2020_7348_MOESM2_ESM.docx]

| **Study** | **Enrolled study** | | | | | | | | **NOS score** |
| --- | --- | --- | --- | --- | --- | --- | --- | --- | --- |
|  | **Selection** | | | | **Comparability** | **Outcome** | | |  |
|  | Representativeness of the exposed cohort | Selection of the non-exposed cohort | Ascertainment of exposure | Demonstration that outcome of interest was not present at start of study | Comparability of cohorts on the basis of the design or analysis | Assessment of outcome | Was follow-up long enough for outcomes to occur | Adequacy of follow up of cohorts |  |
| Gu, Y et al, 2019 | ★ | ★ | ★ | ★ | ★★ | ★ | ★ |  | 8 |
| Huang, L et al, 2019 | ★ | ★ | ★ | ★ | ★★ | ★ | ★ |  | 8 |
| Wu, D et al, 2019 | ★ | ★ | ★ | ★ | ★★ | ★ | ★ |  | 8 |
| Chen, B et al, 2018 | ★ | ★ | ★ | ★ | ★ | ★ | ★ |  | 7 |
| Gu, Y et al, 2018 | ★ | ★ | ★ | ★ | ★★ | ★ | ★ |  | 8 |
| Ye H, et al, 2018 | ★ | ★ | ★ | ★ | ★ | ★ | ★ |  | 7 |
| Li, Y et al, 2018 | ★ | ★ | ★ | ★ | ★★ | ★ | ★ |  | 8 |
| Lu, Y et al, 2018 | ★ | ★ | ★ | ★ | ★ | ★ | ★ |  | 7 |
| Wang, Q et al, 2017 | ★ | ★ | ★ | ★ | ★★ | ★ | ★ | ★ | 9 |
| Hua, F et al, 2017 | ★ | ★ | ★ | ★ | ★ | ★ | ★ | ★ | 8 |

**Supplementary file**

**TABLE S2** NOS score of enrolled studies

*Note.* NOS: Newcastle-Ottawa Scale
